# Environmental Effects on Piezoelectric Sensors Array Signals and a Compensated Damage Imaging Method

**DOI:** 10.3390/ma14226742

**Published:** 2021-11-09

**Authors:** Zhiling Wang, Yongteng Zhong, Jinyu Zhou, Chaoyue Li, Lina Zhong

**Affiliations:** 1 School of Mechanical and Electrical Engineering, Jinling Institute of Technology, Nanjing 211169, China; wzl@jit.edu.cn (Z.W.); zhoujy@jit.edu.cn (J.Z.); lichaoyue@jit.edu.cn (C.L.); 2 College of Mechanical and Electrical Engineering, Wenzhou University, Wenzhou 325035, China; 3 School of Mathematics and Information Technology, Jiangsu Second Normal University, Nanjing 210013, China; zhonglina@jssnu.edu.cn

**Keywords:** piezoelectric sensors array, lamb waves, environmental effects, damage imaging, compensation

## Abstract

Piezoelectric sensors array based damage imaging method as a high resolution source localization algorithm is becoming a promising method in structural health monitoring (SHM) technology. However, the environmental variations could affect the gain-phase of array signal. This paper experimentally evaluates the environmental effects on piezoelectric sensors array, and presents a compensated 2D-MUSIC based damage imaging method for composite structures. Firstly, detailed analysis and comparison discussion about the gain-phase difference of array signal when the environmental parameters change, and the gain-phase changes respect to the environmental parameters could be obtained. Secondly, array error matrix is structured and substituted into the steering vector of the original 2D-MUSIC algorithm to compensate. Finally, the compensated 2D-MUSIC algorithm is applied for estimating the initial estimates of damage. After substituting these initial estimates, the cost function is minimized by adaptive iterative calculating the reasonable location of the damage source. The experiments on an epoxy laminate plate demonstrate the validity and effectiveness of the proposed method.

## 1. Introduction

Structural health monitoring (SHM) is perceived as a significant method for determining the integrity of structures since it could provide an early indication of physical damage [1,2]. Lamb wave-based imaging method has proved to be a promising damage monitoring technology and has been widely researched in the field of structural health monitoring of composite structures [3,4].

In recent years, compact piezoelectric sensor array based damage imaging method as a high resolution source localization algorithm becomes a promising method in structural health monitoring (SHM) technology. Wang et al. [5] presented a Lamb wave and linear piezoelectric lead zirconate titanate (PZT) array-based monitoring method for the detection and quantification of crack damage on a T6061 aluminum plate. Huan et al. [6] proposed an SH0 wave linear array SHM system which can detect a through-thickness hole as small as 2 mm of a plate. Tian et al. [7] configured a compact linear fiber Bragg grating phased array and implemented an adaptive phased array algorithm which can precisely locate damage in plates. Ren et al. developed a scanning spatial-wavenumber of guided wave-based imaging method for multiple damages [8] and multi-impact [9] on aircraft composite structures. The authors combined the near-field sampling phased-array damage monitoring algorithm and the two-dimensional phased array for developing a phased array damage imaging method [10]. Multiple signal classification (MUSIC) algorithm has been promoted in impact signal localization stiffened composite structures due to its high accuracy and wonderful imaging resolution [11]. Zhong et al. [12] developed an improved sensor array with two additional sensors above and below the linear sensor array, and presented time difference and two-dimensional multiple signal classification (2D-MUSIC) based impact localization for omni-directional localization on composite structures.

However, environmental parameters like temperature, vibration, and load may change the phase and amplitude of Lamb wave and will lead to false damage imaging. Therefore, many researchers have experimentally evaluated the effect of environmental conditions on Lamb wave signals [13]. Ren et al. adopted Gaussian mixture model to characterize the environmental conditions cased uncertainty on guided wave signals and measured its variation during the damage monitoring process [14], and proposed delay-and-sum algorithm based multi-damage imaging method under environmental conditions [15]. Sun et al. [16] presented a compensation method for Lamb wave-base damage detection within a non-uniform temperature field. Mohabuth et al. [17] investigated the effect of applied or thermally induced stresses on Lamb wave propagation. Yang et al. [18] investigated online and offline monitoring of composite bolted joints under tensile load using piezoelectric transducers. However, the current works focused on developing compensation strategies for the effects of environmental condition on Lamb wave using a single pitch–catch path, which are unavoidable in piezoelectric sensors array real application.

With the aim to solve the environmental effects on array signal, a compensated 2D-MUSIC-based damage imaging method for composite structures is introduced in this paper. This paper is organized as follows: Section 2 introduces the process of sensors array based damage imaging method. In Section 3, the environmental effects experiments on composite structures are researched by experiments. Eventually, Section 4 discusses the damage location compensation results, and Section 5 gives the conclusion.

## 2. Sensors Array Based Damage Imaging Method

### 2.1. Piezoelectric Sensors Array Based Damage Imaging Method

**Figure 1:**
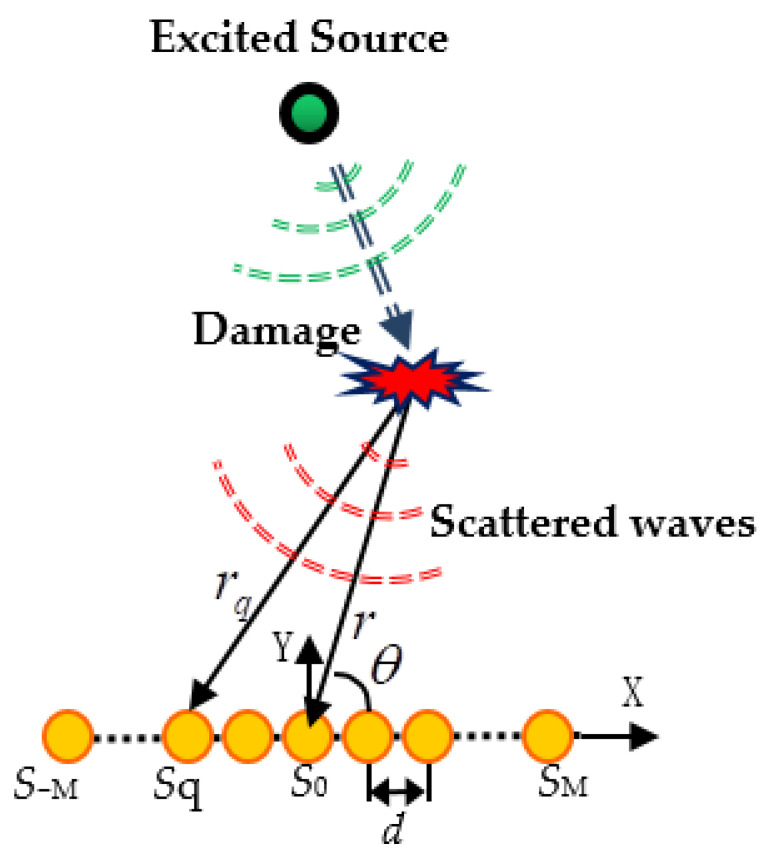
Damage scattered signal model.

In this paper, the observation signal consists of a uniform linear array of 2M+1 piezoelectric sensors with a space of *d* as shown in Figure 1. Assuming the scattered waves induced by damages with a certain frequency of $\omega_{0}$, PZT*q* under the near-field situation can be presented as

(1) $$x_{q}(t)=\frac{r}{r_{q}}s(t)e^{-j\omega_{0}\tau_{q}}+n_{q}(t),\;q=-M,\cdots ,0,\cdots ,M$$

where $n_{q}(t)$ express the background noise, $\omega_{0}$ denotes the center frequency of scattered source, c is the Lamb wave velocity. The distance between impact source and PZT q is calculated as

(2) $$r_{q}=\sqrt{r^{2}+d^{2}{\left(q-1\right)}^{2}-2rd(q-1)\cos\theta }$$

The scattered signal arriving time difference between PZT*q* and PZT0 is defined as

(3) $$\tau_{q}=(r-r_{q})/c$$

The steering vector of PZT q is denoted as

(4) $$a_{q}(r,\theta )=\frac{r}{r_{q}}\exp(j\omega_{0}\tau_{q})$$

For the whole array response signals can be presented as

(5) $$X(t)=A(r,\theta )x_{0}(t)+N(t)$$

where,

$$\begin{array}{l} X(t)={\left[x_{-M}(t),\cdots ,x_{0}(t),\cdots ,x_{M}(t)\right]}^{T}\\ A(r,\theta )={\left[a_{-M}(r,\theta ),\cdots ,a_{0}(r,\theta ),\cdots ,a_{M}(r,\theta )\right]}^{T}\\ N(t)={\left[n_{-M}(t),\cdots ,n_{0}(t),\cdots ,n_{M}(t)\right]}^{T} \end{array}$$

### 2.2. Environmental Conditions Effects Compensation Method

The standard 2D-MUSIC method in ref. [19] is used to estimate the distance and direction without considering the environmental effect. However, the signal gain-phase changes of array can lead to the actual steering vector unequal to signal subspace when environmental parameters vary. According to the actual steering vector of array sensor signal is hardly orthogonal to noise subspace bring about the accuracy of damage localization in spatial spectrum declined. Thus this problem is resolved by compensating steering vector.

Suppose Γ means gain-phase errors in steering vector due to environmental effect, and gain-phase errors matrix can be denoted as

(6) $$\Gamma (r,\theta )=diag[\Gamma_{-M}(r,\theta ),\Gamma_{0}(r,\theta ),\cdots ,\Gamma_{M}(r,\theta )]$$

The gain-phase error of the *q*th PZT under environmental parameters variation is

(7) $$\Gamma_{q}(r,\theta )=C_{q}\exp[j\omega_{0}({\hat{\tau }}_{q}-\tau_{q})]=C_{q}\exp(j\omega_{0}\Delta \tau_{q}),q=-M,\cdots ,0,1,\cdots ,M$$

where $C_{q}$ and $\Delta \tau_{q}$ are the signal amplitude and phase errors of PZT*q* when environmental parameters vary. ${\hat{\tau }}_{q}$ denotes the actual arriving time difference of scattered signal between PZT*q* and PZT0.

The signal steering vector after compensated is performed as

(8) $$\tilde{A}(r,\theta )=\Gamma (r,\theta )A(r,\theta )$$

By substituting Equation (8) into Equation (5), the actual scattered source location can be estimated. 

The covariance matrix of whole response signals is

(9) $$\tilde{R}=\frac{1}{N}XX^{H}=U_{S}\Sigma_{S}U_{S}^{H}+U_{N}\Sigma_{N}U_{N}^{H}$$

where $U_{S}$ and $U_{N}$ mean the signal subspace and the noise subspace corresponding to the largest eigenvalue $\Sigma_{S}$ and the small eigenvalues $\Sigma_{N}$, *N* denotes the sampling length, *H* is the conjugate transpose.

The spatial spectrum can be computed as

(10) $$P_{\mathrm{MUSIC}}(r,\theta )=\frac{1}{{\tilde{A}}^{H}(r,\theta )U_{N}U_{N}{}^{H}\tilde{A}(r,\theta )}$$

where the spatial spectrum peak point is considered as the initial estimate value of damage location for adaptive iterative method. Construct a cost function J in ref. [20], the correct estimates of (r,θ) could be acquired by minimizing the cost function.

## 3. Environmental Effects Experiments on Composite Structures

**Figure 2:**
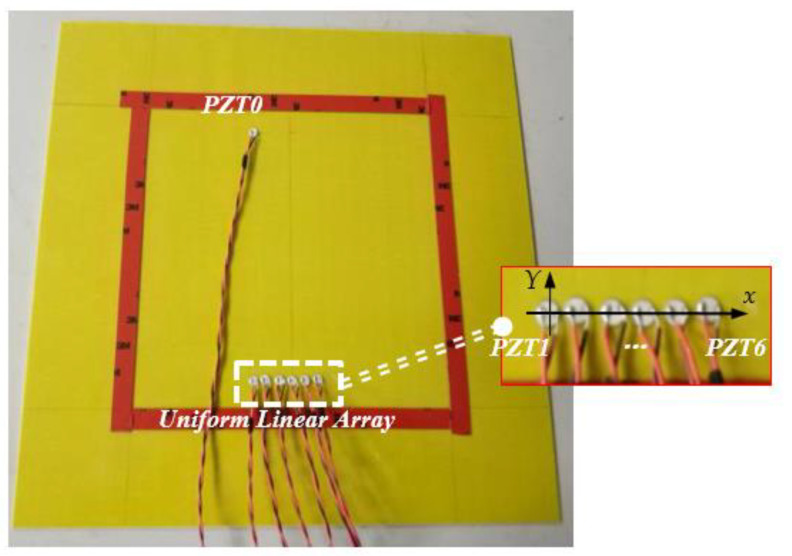
Overall view showing the plate, active sensor PZT0, and an uniform linear array.

The series of experiments were carried out in order to investigate the environmental condition effect on piezoelectric sensors array based damage imaging method. As seen in the Figure 2, the environmental experiments were carried on an epoxy laminate plate with a dimension of 400 mm × 400 mm, and the ply sequence is [02/904/02]_S_, the thickness of each ply is 0.125 mm. A uniform linear array (ULA) is bonded on the epoxy laminate plate surface, which consists of 6 PZTs and are labeled as PZT1, PZT2..., PZT6 respectively from the left to the right, and the distance between adjacent PZT center is 1 cm. Another single PZT upon the array is labeled as the excitation sensor PZT0 to simulate the scattered waves induced by damages, and the distance between PZT0 and PZT1 is 250 mm.

### 3.1. Temperature

**Figure 3:**
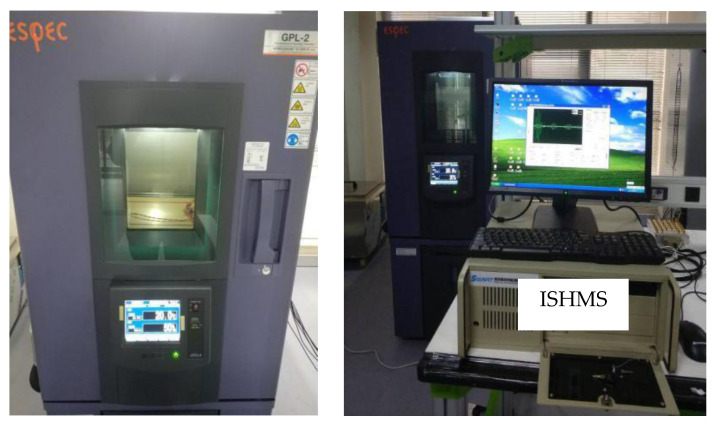
Schematic of experimental setup for temperature testing.

Among various environmental condition, temperature variations is substantially limiting Lamb waves propagating in the thin plate. The effect of temperature variations firstly experimentally evaluated, and the experimental setup is shown in Figure 3, including a thermostat, an epoxy laminate plate and an integrated structural health monitoring scanning system (ISHMS). The epoxy laminate plate was placed on shelf in the thermostat, and the wires through the hole of rubber plug and connect ULA PZT sensors. The ISHMS is developed to control the excitation and sensing of the PZT sensor array.

**Figure 4:**
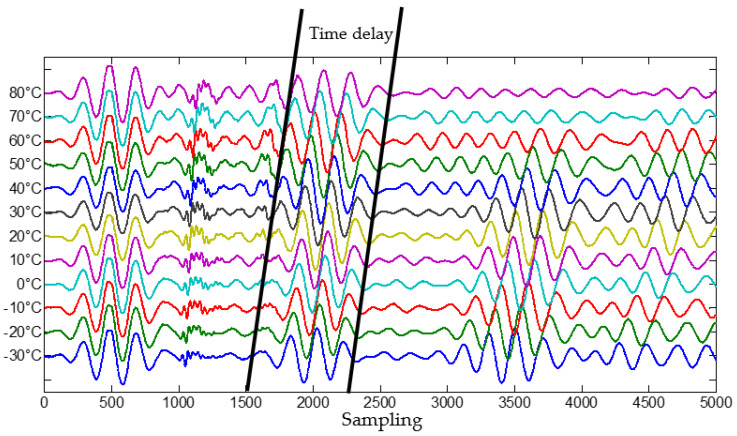
Experimental received Lamb wave array signal collection from −30° to 80°.

**Figure 5:**
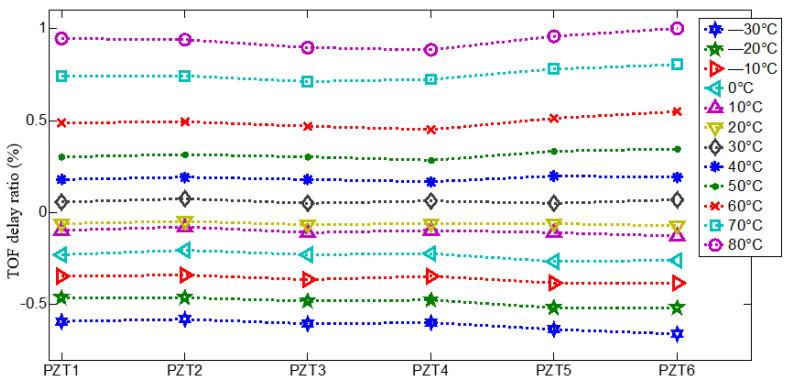
TOF delay ratio of each PZT at −30° to +80°.

The received Lamb wave array signal at −30 to +80° at the frequency of 50 kHz are shown in Figure 4. It can be observed that the times of flight (TOF) of Lamb wave present an obviously delay when the temperature vary from −30° to +80°, and the amplitude of Lamb wave decreases with increase temperatures. Therefore, the temperature delays the TOF of Lamb wave which will result in false the scattered signal arriving time difference in Equation (3). By comparing the TOF of Lamb wave propagation at a normal temperature, TOF delay ratio of each array element vs. temperature, PZT 1–PZT 6, could be obtained which shown in Figure 5. It shows that the time delay of each PZT is almost proportion to temperature increasing, and the time delay induced by temperature could be denoted as weights vector, that is the gain-phase errors matrix in Equation (6).

### 3.2. Vibration

**Figure 6:**
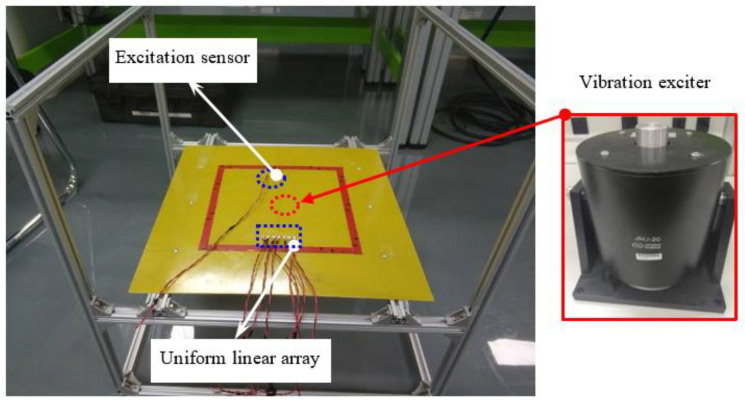
Schematic of experimental setup for vibration testing.

The schematic of experimental setup for vibration testing is shown in Figure 6. The working frequency range of vibration exciter is 1 Hz to 1600 Hz. The vibration exciter loaded at the center of the plate under four edges clamped condition. The sizes of epoxy laminate plate, sensor arrangements and systems parameters in ISHMS are same as the frontal experiment.

**Figure 7:**
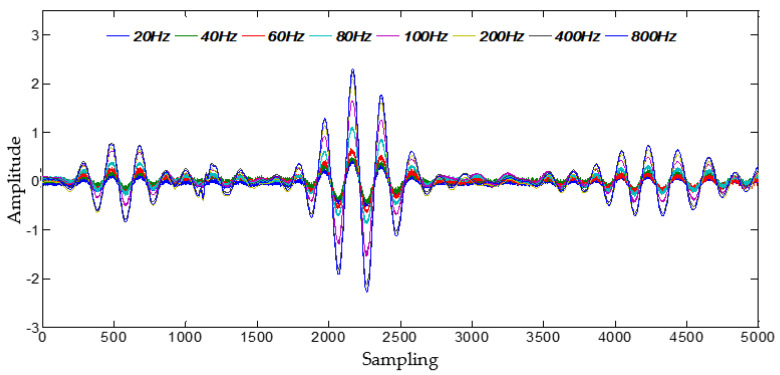
Comparison of PZT 1 sensor signals with vibration at different Hz frequency.

**Figure 8:**
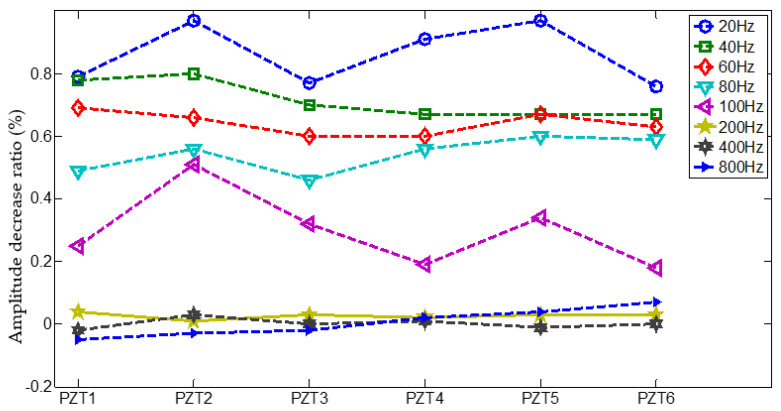
Extracted amplitude decrease ratio comparing with the signal in static condition.

A comparison of PZT 1 sensor signals with vibration at different Hz frequency are given in Figure 7. As seen in figure, it can be observed that for all sensor signals with vibration at different Hz frequency, the time of flight (TOF) of Lamb wave fluctuate slightly as the vibration frequency varies, but the amplitude of Lamb wave decrease at higher Hz vibration. More details about the extracted amplitude decrease ratio comparing with the signal in static condition are shown in Figure 8. It is clear in figure that the amplitude of each of PZT sensor signal at high frequency (200–800 Hz) almost remained intact, but at low frequency (20–100 Hz) decreased significantly, and the amplitude of each of PZT sensor signal changed inconsistently. Therefore, the amplitude decrease ratio could be denoted as weights vector induced by vibration, that is the gain-phase errors matrix in Equation (6).

### 3.3. Load

**Figure 9:**
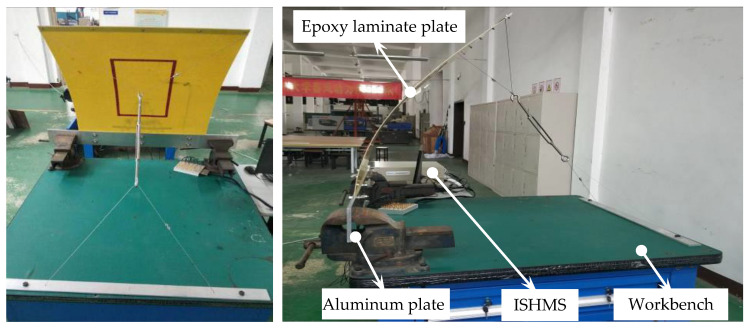
Schematic of experimental setup for Load testing.

The schematic of experimental setup for Load testing is shown in Figure 9. A deformation system is designed to apply load, and the average curvature of the plate denotes different loading conditions. The average curvatures of the plate corresponding to deformation degrees from 1 to 9 were 2.41, 2.31, 2.21, 2.16, 2.06, 2.02, 1.96, 1.91, and 1.83 rad/m, respectively.

**Figure 10:**
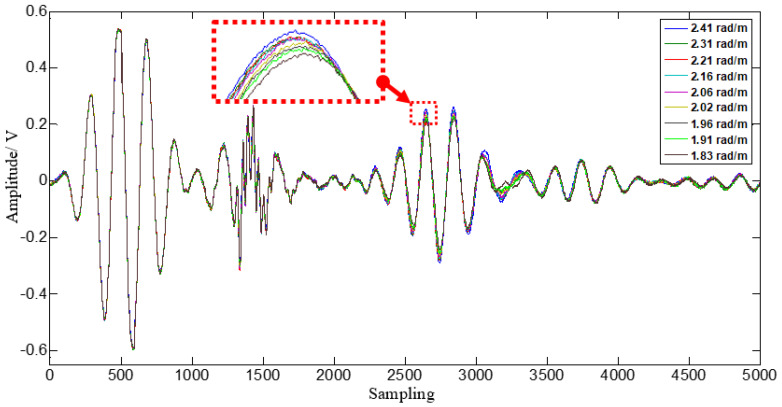
Comparison of PZT 1 sensor signals with different loading conditions.

**Figure 11:**
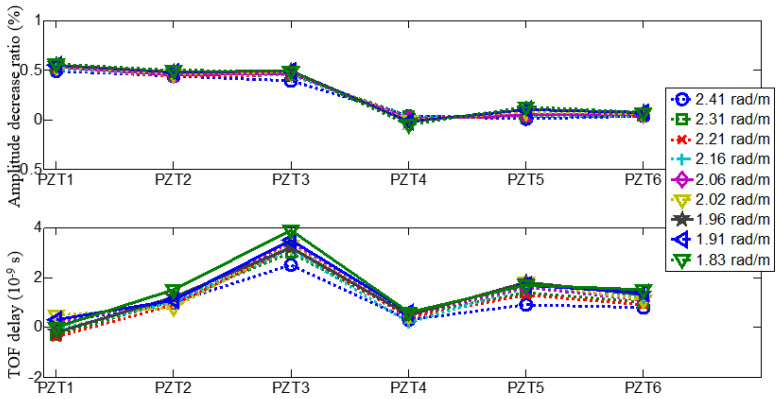
Schematic of experimental setup for load testing.

A comparison of PZT 1 sensor signals with different loading conditions is given in Figure 10. It can be observed from figures that their shape does not change significantly when applied load changes. The amplitudes of each PZT signal increase and the TOF decrease as the load increases. The amplitudes and TOF magnitude of each PZT of sensor array change are different due to the different propagation path. Here, we defined the gain-phase errors contrast caused by loading and demonstrated in Figure 11.

## 4. Damage Imaging Compensation Results and Discussion

**Figure 12:**
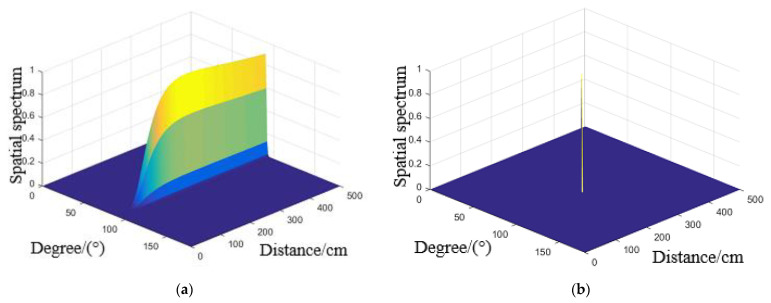
Spatial spectrum estimated for 50 °C by (**a**) Stander 2D-MUSIC (**b**) Compensated 2D-MUSIC.

**Figure 13:**
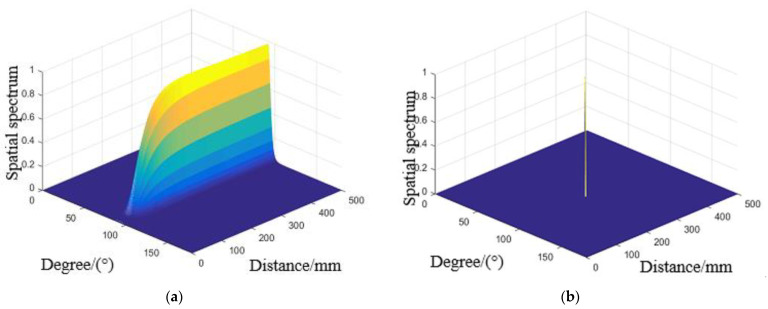
Spatial spectrum estimated for 600Hz by (**a**) Stander 2D-MUSIC (**b**) Compensated 2D-MUSIC.

**Figure 14:**
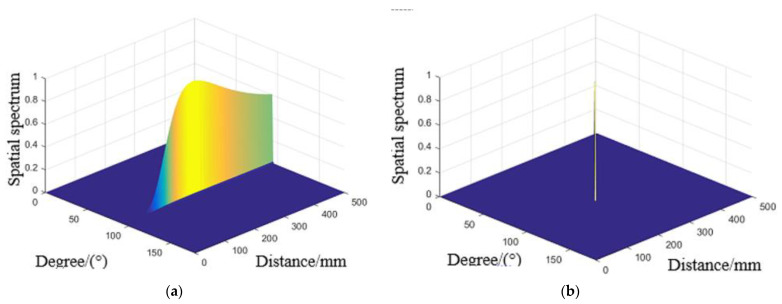
Spatial spectrum estimated for 2.41 rad/m by (**a**) Stander 2D-MUSIC (**b**) Compensated 2D-MUSIC.

To verify the method proposed for damage location under different environmental effects in this paper, the cases studied including temperature, vibration and load. According to the direct wave signals of each array element in above Figures, input MUSIC method to calculate the steering vector A(r,θ) of the array signal, and the array error matrix obtained from environmental effects experiments were substituted into its steering vector, which estimate the azimuth (r,θ) of the scattered signal source. At the same time, the spatial spectrum peak obtained by the proposed method is substituted into the adaptive iterative method as the initial value to compensate and enhance the imaging resolution. The typical spatial spectrum at simulated damage (250 mm, 90°) at 50 °C, 600 Hz and 2.41 rad/m obtained are shown in Figure 12a, Figure 13a and Figure 14a. And the compensated spatial spectrum under different environmental effects are shown in the Figure 12b, Figure 13b and Figure 14b comparison between the stander 2D-MUSIC and MUSIC after compensation.

By comparison of the spatial spectrum estimated by standard 2D-MUSIC and compensated 2D-MUSIC, piezoelectric sensors array based damage imaging method has a better localization result after considering the environmental effects. All the results of three environmental effect cases and their errors estimated by stander 2D-MUSIC, and the proposed method are summarized in Table 1. The maximum estimation error in distance and direction is 1.9 cm and 4° respectively under different temperatures. The maximum estimation error in distance and direction is 2.4 cm and 4° respectively under different vibrations. The maximum estimation error in distance and direction is 1.0 cm and 4° respectively under different load. The experimental results that the proposed method is successfully applied to detecting damage under different environmental factors varies.

## 5. Conclusions and Future Works

Lamb waves based methods were reported to be very effective in detecting and localizing defects in composite structures. However, the performance of these techniques is susceptible to environmental conditions. In this paper, a piezoelectric ultrasonic array method, based on two-dimensional multiple signal classification (2D-MUSIC) algorithm and adaptive sensor array error calibration, is proposed for damage localization on a composite plate under variable environmental conditions. Firstly, the observed data modal considering imprecise gain-phase signals in sensor array induced by variable temperature environments is built using the 2D-MUSIC algorithm. Secondly, the observed signal model of the sensor array is represented by error calibration matrix with unknown gains and phases, and it used to construct the cost function including sensor array parameters. Finally, the cost function is minimized by adaptive iterative for calculating the sensor array error parameters and the exact location of the scattered damage source. The experiments show that the impact damage localization results estimated by the proposed method have more accurate and higher imaging resolution than standard 2D-MUSIC method under environmental condition. The maximum estimation error in distance and direction is 1.9 cm and 4° respectively under different temperatures. The maximum estimation error in distance and direction is 2.4 cm and 4° respectively under different vibrations. And the maximum estimation error in distance and direction is 1.0 cm and 4° respectively under different loads.

However, the only single environmental effect is investigated in this paper. Further research is still worthy to address systematically the effects of simultaneous environmental factors on the proposed method. Besides, detailed research also needs to be performed on real structural damage in future research.

## Figures and Tables

**Table 1:** Localization results under environmental conditions using the standard and compensated 2D-MUSIC.

Environmental Factors		Standard 2D-MUSIC		Compensated 2D-MUSIC	
		${\overset{⌢}{r}}_{s}$$/E_{s}^{r}$ (mm)	${\overset{⌢}{\theta }}_{s}$$/E_{s}^{\theta }$ (°)	${\overset{⌢}{r}}_{c}$$/E_{c}^{r}$ (mm)	${\overset{⌢}{\theta }}_{c}$$/E_{c}^{\theta }$ (°)
Temperature/°C	−20	242/8	95/5	242/8	93/3
	0	233/17	96/6	233/17	94/4
	10	289/39	96/6	289/19	94/4
	20	231/19	96/6	240/10	94/4
	50	248/2	95/5	248/2	93/3
	70	246/4	95/5	249/1	93/3
Vibration/Hz	30	242/8	94/4	244/6	94/4
	60	257/7	94/4	257/7	93/3
	90	278/28	94/4	274/24	93/3
	300	238/12	92/2	241/9	92/2
	600	234/16	91/2	238/12	90/0
	900	208/42	91/3	246/4	90/0
Curvature induced by Load/rad/m	2.31	258/8	93/3	255/5	93/3
	2.21	244/6	94/4	245/5	94/4
	2.16	242/8	94/4	244/6	93/3
	2.06	266/14	93/3	248/2	92/2
	1.91	240/10	94/4	242/8	94/4
	1.83	260/10	93/3	260/10	93/3

## Data Availability

The data that support the findings of this study are available from the corresponding author upon reasonable request.
